# External Use of Chloroform in Lumbago

**Published:** 1849-06

**Authors:** 


*External use of Chloroform in Lumbago.*—In the *Gazette des Hopitaux*, of October, 1848, it was announced that Dr. Moreau, of Tours, had discovered that chloroform, applied to the skin over the seat of the disease, had proved very efficacious in the treatment of lumbago.

Numerous experiments made since that time, both in France and elsewhere, with this most astonishing agent, have very conclusively established the efficacy of its external use, not only in lumbago, but in numerous other nervous diseases. No later than yesterday, a professional friend of this city related to me a case of the most distressing facial neuralgia which had been immediately and permanently relieved by a single application of chloroform. The same gentleman has used it with equal success in neuralgic pains in other parts of the body. I have myself used it with entire success in two cases of the most intolerable earache.

A brief account of some of the cures reported by Dr. Moreau may prove interesting:

1. Lapeniere, epileptic, aged 19 years, of lymphatic temperament, was seized about three weeks ago for the first time, with lively pains in the loins. The pains soon became intolerable, and even extended into the left thigh. A large piece of batting, moistened with chloroform, was applied *loco dolenti*. The pain disappeared entirely in the course of twenty-five minutes, and has not since reappeared.

2. A man, aged 59 years, who had suffered from repeated attacks of articular rheumatism, was suddenly seized, on the evening of the 21st ult., with a most violent lumbago. The pain was felt principally in the left loin and hip. The least change of position or movement produced the most excruciating suffering; difficulty of respiration excessive; chilliness, general perspiration; considerable febrile reaction; tongue loaded. On the morning of the next day chloroform was applied in the usual manner. Relief obtained in twenty or thirty minutes.

The above cases are very similar to those which were reported in a preceding number of the Gazette. The third case, the termination of which was equally satisfactory, is of greater value than either of the others, from the fact that the pains to which the patient was very subject, almost invariably lasted from fifteen days to a month. Under the use of chloroform he was completely relieved of them in three days.

3. M. Trubin, of robust habit, but subject for many years past to rhumatismal pains, the seat of which was especially in the region of the kidneys, was seized with one of these pains, which was very intense, and occupied the right lumbar region, from whence it reached to the hip and thigh of the same side. The slightest movement augmented it very greatly; the patient walked with great difficulty, dragging his leg. Pressure produced no pain at any point. Twenty-four hours after the appearance of the disease, chloroform was applied over the seat of the pain. Its usual effects were produced. The patient experienced a smarting sensation, and the skin reddened. In the evening a very sensible amelioration had occurred. The next day the pain had disappeared; there only remained a feeling of numbness and weight in the regions which the evening before had been painful. The next day, the third from the first application of the chloroform, the cure was complete. The skin desquamated over the points touched by the chloroform.

We may here make a remark which is of some importance in a therapeutical point of view. It is necessary to act promptly and with vigor; this is the price of success. The more recent the lumbago the greater are the chances of cure. It is equally important not to be sparing with the chloroform. M. Moreau seldom employs less than from one to two ounces; and he also contrives that the batting shall cover as nearly as possible the whole of the painful part. After the application of the chloroform, the patient must be put to bed and covered in such a way as to protect him almost entirely from the action of the anæsthetic vapors. The unsatisfactory result of the following case may, we believe, be attributed to the quantity of chloroform employed being too small:

4. Ardan, aged 30 years, had never suffered pains of any description, until on the 25th of October, when he was suddenly seized with intense pains in the loins; he was bent double, without power to straighten himself; the pain was felt most between the shoulders. On the morning of the 28th, an application was made along the course of the vertebral column of batting, upon which had been poured at most about 50 drops of chloroform, and which was allowed to remain only about ten minutes—M. Moreau wishing to avoid producing vesication, which had occurred in a case that we will presently report. The pain was almost instantly calmed, but it reappeared in the evening, though less distressing than it was in the morning. The patient, moreover, complained of excessive heat in the part with which the chloroform had been in contact. The next day the pains were very slight, but they did not entirely disappear until two days later.

The rapidity with which the pain yields has always appeared to be in proportion to the extent of the injury done the skin, and that a slight and partial vesication, disseminated here and there has been produced. The contrary is the case when the chloroform has only determined a more or less vivid redness. In the following case there was complete vesication and very rapid recovery:

5. Favier, aged 55 years, has suffered towards autumn, for many years, with rheumatismal pains, sometimes articular, sometimes muscular, of which the mean duration was from six weeks to two months. The patient remembers to have had his first attack of lumbago at the age of eighteen years. The vague and sometimes very acute pains that he feels in different parts of the body, alternate ordinarily with violent pains in the head.

On the 3d of November, attacked with lumbago, the intensity of which rapidly increased and prevented all motion. On the 11th, chloroform was applied in the usual manner. Favier first complained of an icy coldness in the part to which the chloroform had been applied; then soon after a sharp and growing heat, which he compared to that of a red iron. The apparatus was removed at the expiration of twenty minutes, when the skin was of a crimson red. The pains had disappeared as if by enchantment. The next day the patient got up, walked about, went up and down stairs without exciting any pain. The chloroform, in this instance, produced a true vesication; the epidermis was raised and filled with serum. Four days after, the patient still felt in the parts a sensation of heat and numbness.

The curative action of chloroform is not always equally prompt and instantaneous. This, however, is only the exception to the rule, for in two cases only out of seven was it necessary to renew, after a considerable interval, the application of the remedy.

6. B. has been subject, for three or four years past, to rheumatismal pains, which, two or three times a year, manifest themselves in the left shoulder. These pains appeared in the early part of December; it was impossible for B. to move his arm without crying out; the pain extended to the finger points. Blisters and cups were applied without affording any relief. The diseased shoulder was then enveloped in batting saturated with chloroform; the pains yielded, but slowly; nevertheless, B. was able to resume his work the same day. The next day the pains reappeared; another application of the chloroform overcame them in less than thirty minutes.

7. M. Malson, director of the hospital, has been subject to lumbago for a great number of years.

On the 13th of Dec., an intense pain arose in the lower part of the loins; it was continuous, with frequent twitchings; the slightest movements were impossible; the patient was obliged to keep his bed. Frictions, with a complicated liniment, sinapisms, and injections of turpentine were of no avail. On the 16th, chloroform (about one ounce and a half) was employed. To a sensation of burning, which was easily borne, succeeded a quiet, agreeable heat. At the end of eight or ten minutes the patient was able to turn himself in bed without exciting any pain. The chloroform not appearing to act with its customary energy, the batting was allowed to remain for half an hour. In the evening the patient got up, dressed himself, walked about in his chamber, and to his office, without experiencing anything save a slight pricking sensation in the loins, similar to that produced by the application of sinapisms. About six o'clock, however, the pains returned, though much less intense than the first, dull, and without anything twitching or shooting in their character. The night was passed in this condition, and on the morning of the next day the pains had assumed a certain violence. Chloroform reapplied. The skin scarcely presented the redness produced by a slight sinapism, when, in an instant, as was the case in the first instance, all pain ceased, and the patient was repossessed with entire freedom of motion. The next day the patient felt a certain stiffness in the loins, numbness, etc. The same day he set out for Paris in a carriage, remained absent for many days; no suffering has been felt since. The last consideration that I shall offer upon this subject relates to a neuralgic affection other than lumbago. M. Moreau employed chloroform in a single case of sciatica and failed. He has, nevertheless, communicated the outlines of the case, which contain certain peculiarities of a nature to induce us to believe that chloroform was not entirely innocent of the cure which was obtained in less than twelve days.

8. Madame F., a woman employed in the hospital, had suffered for some days with a very acute sciatic pain. The disease had commenced by a lumbago, induced in consequence of a strain. The first application of chloroform only very slightly allayed the pains; the second produced no effect whatever; nevertheless, the liquid had produced a large erythematous patch upon the skin. The next day the hip and the superior part of the thigh were covered by a large blister, the surface of which, after the removal of the epidermis, was touched at different points with the batting saturated with chloroform; a little while after this the pains had disappeared. In the night, the pains shot about anew. The next day the patient got up and walked about. It was still, however, not until seven or eight days later that she was completely relieved and able to resume her work.

The mechanism of the action of chloroform, used in this way, appears to be this:—There is first rubefaction, and then revulsion produced by this rubefaction of the skin; afterwards, absorption and direct action of the anæsthetic upon the muscular and tegumentary extremities of the nerves. D. W. Y.

